# Protecting yourself and your patients from COVID-19 in eye care

**Published:** 2020-03-30

**Authors:** Victor H Hu, Elanor Watts, Matthew Burton, Fatima Kyari, Ciku Mathenge, Fatemeh Heidary, Jeremy Hoffman, Elmien Wolvaardt

**Affiliations:** 1 Clinical Assistant Professor, International Centre for Eye Health, LSHTM^i^ & Consultant Ophthalmologist: Mid Cheshire Hospitals; 2 Junior doctor & MSc Public Health for Eye Care student, LSHTM^i^; 3 Professor of International Eye Health and Director, International Centre for Eye Health, LSHTM^i^; 4 Associate Professor: International Centre for Eye Health, LSHTM^i^ & Consultant Ophthalmologist: University of Abuja^ii^; 5 Consultant Ophthalmologist & Director of Training and Research, Rwanda International Institute of Ophthalmologyiii; 6 Head of Ophthalmology Division, Ahvaz Jundishapur University of Medical Sciences^iv^; 7 Clinical Research Fellow: International Centre for Eye Health, LSHTM^i^; 8 Editor: *Community Eye Health Journal*, International Centre for Eye Health, LSHTM^i^

**The COVID-19 pandemic is having profound repercussions around the world.^1^,^2^ Eye health care providers, along with other health services, are having to work out how care should now be delivered.**

**Figure:**
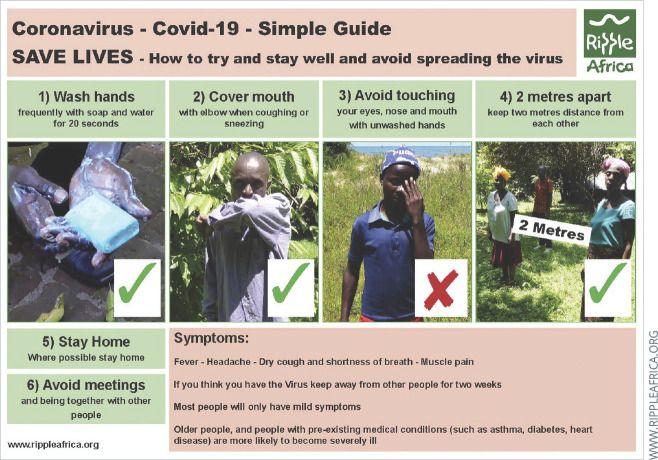
Educating the public about COVID-19 is an essential component of interventions to stop the spread of the novel coronavirus. MALAWI

An ophthalmologist working in Wuhan, China, was among the first to recognise the possible emergence of a new respiratory disease outbreak.^3^ Sadly, he and a number of his colleagues subsequently died from the infection. It is now understood that severe acute respiratory syndrome coronavirus 2 (SARS-CoV-2), the virus that causes Coronavirus Disease 2019 (COVID-19), is transmitted by droplets, aerosol particles, human-to-human contact or via fomites (particles of skin cells, hair, clothing and bedding). Infection can occur when viral particles enter the mouth, nose or eyes.^4^

To help stop the spread of infection, some measures have been implemented in many countries. These include:

- Self-isolating measures, i.e., staying at home.^5^ This is particularly important for people considered to be vulnerable, i.e. more at risk of severe COVID-19 complications. They include older people, those with underlying medical problems (e.g., diabetes and chronic respiratory, heart, kidney, neurological and liver diseases), people who are immunosuppressed, and people with marked obesity (body mass index [BMI] > 40).^6^ Many countries are currently asking their whole population to stay at home, with the exception of key workers and essential activities, to limit the spread of the virus and the number of people likely to need intensive care.
- Social distancing. If people do have to go outside of the home, they are advised to keep a distance of at least 2 metres from others.^7^
- People who might have had a travel history or possible contact with an infected person are asked to self-isolate or are quarantined.^8^

Health care workers, especially in a hospital setting, are on the front line in the efforts against this pandemic.^9–11^ There is considerable concern in the ophthalmic community about the risk of infection as there is increasing information that the initial infectious dose (amount of virus transmitted) and resulting high viral load may increase the risk of severe COVID-19 disease.^12^,^13^

There are a number of guidelines on how to adapt services and adjust practice to limit the spread of infection in a clinical setting; however, the evidence is still limited. This uncertainty, the many differences between countries and individual clinical settings, and ongoing practical challenges such as shortages in personal protective equipment (PPE) means that things will be changing all the time and eye health workers will have to adapt and make the best of what is available to them at the time.

Guidelines are also changing constantly to reflect new research as it comes in. We encourage you to visit the websites given in the panel to receive further updates. The Centers for Disease Control (CDC) provides guidance for dealing with PPE shortages at **https://bit.ly/PPEguide**.

**Guidelines and useful websites**

- **World Health Organization (WHO). Country & Technical Guidance – Coronavirus disease (COVID-19)** ^14^
- **World Health Organization (WHO). Rational use of personal protective equipment for coronavirus disease (COVID-19)** ^15^
- **International Agency for the Prevention of Blindness. COVID-19 Resources: Here is what we know** ^16^
- **International Council for Ophthalmology. Coronavirus Information for Ophthalmologists** ^17^
- **Royal College of Ophthalmologists COVID-19 Clinical Guidelines** ^18^
- **American Academy of Ophthalmologists. Coronavirus and Eye Care** ^19^
- **World Council of Optometry. Statement concerning COVID-19** ^20^
- **Centers for Disease Control (US) Strategies to Optimise the Supply of PPE** ^21^

The particular context of an eye health service will influence how the guidelines can be put into practice, such as the availability of personal protective equipment (PPE), access to emergency care for known COVID-19 patients, and the available means of communication.

The current guidelines can be summarised as follows:

1. Cease providing any routine eye services other than urgent or emergency care
2. Protect health care workers
3. Reduce transmission to other patients, and between patients and health care workers

## 1. Cease providing any routine eye services other than urgent or emergency care

Postpone or defer any non-urgent eye appointments. Patients coming to an eye clinic or eye unit are at risk of exposure to COVID-19 infection, and many patients attending for eye care are elderly or suffer from underlying chronic medical conditions such as diabetes; they are at increased risk of severe COVID-19 and social distancing is therefore particularly important for them.

Also, reducing eye care services can release health workers to be deployed to other areas of health care where they may be able to play an important role during the pandemic.

Where there is an appointment system in place, defer pre-existing appointments by text message/phone message, letter or other means, as available. Conducting a telephone consultation may be helpful to give advice or, if necessary, decide whether a face-to-face review is essential. When issuing a prescription, follow local guidelines. Ideally, send patients written information about any appointment deferrals, the advice given and how new appointments will be made once the situation allows. If possible, set up pathways for urgent/emergency care together with measures to reduce transmission, as discussed in this article. Tell patients how they can gain access to such care.

### Patients needing urgent or emergency care

There will be patients who need to be seen and treated urgently. Services should continue to be provided, where possible, to patients at high risk of visual loss without treatment. This may include people with the conditions such as these^18^,^22^:

- Exudative age-related macular degeneration
- Severe diabetic retinopathy
- Acute retinal detachment
- Advanced or rapidly progressive glaucoma
- Severe, active uveitis
- Serious ocular oncology conditions
- Retinopathy of prematurity (screening and treatment)
- Globe rupture or other significant trauma
- Serious ocular infections (microbial keratitis, endophthalmitis)

Whether it is appropriate for patients to be seen for urgent/emergency eye care will depend on the individual patient, the risk of significant harm if treatment is delayed and the situation of the eye care provider. Having only one seeing eye would be a strong factor in favour of a patient being seen, for example. If possible, patients should stay away from eye health care settings if they are older than 70 years, have serious pre-existing health problems, or are immunosuppressed.

Table 1 gives some guidance on triaging patients and the appropriate precautions to take.

**Table 1:** AAO interim guidance on ophthalmology patient triage and precautions^19^

Clinical situation	Patient management / precautions
1. Routine ophthalmic issues and previously scheduled appointments	Routine problems should be deferred, and previously scheduled appointments should be cancelled Appointments should be rescheduled only upon clearance from public health authorities Refill all necessary medications
2. Urgent ophthalmology appointment for a patient with no respiratory illness symptoms, no fever, and no COVID-19 risk factors	Standard precautions* Added precaution of not speaking during slit-lamp biomicroscopic examinations is appropriate In the setting of adequate PPE supplies, use of surgical mask and eye protection** for the clinician as well as surgical mask for the patient may reduce asymptomatic and pre-symptomatic transmission
3. Urgent ophthalmic problem in a patient with respiratory illness symptoms, but no fever or other COVID-19 risk factor	The patient can be seen in the eye clinic The patient should be placed in an examination lane immediately with the door closed and placed in a surgical mask. The treating ophthalmologist and health care personnel require surgical masks at minimum Gown, gloves, surgical mask and eye protection are recommended for the clinician.† An N-95 mask should be worn if a procedure is planned that will result in aerosolized virus The examining room must be disinfected after examination
4. Urgent ophthalmic problem in a patient who is at high risk for COVID-19	The patient is best sent to the ER (emergency room) or other hospital-based facility equipped to evaluate for, and manage, COVID-19 If the patient has an urgent eye problem based on screening questions, the facility should be one that is equipped to provide eye care in the hospital setting If SARS-CoV-2 infection is confirmed, CDC (or hospital) guidelines for care of suspected COVID-19 patients should be followed for **health care facility preparation** and **infection control** Eye care is best provided in the hospital setting. Transmission precautions‡ for treating ophthalmologists include wearing a surgical mask, gown, gloves and eye protection (face shield or goggles, if available)
5. Urgent ophthalmic problem in a patient with documented COVID-19 (or person under investigation [PUI])	The patient should remain in the hospital setting if possible Determine whether the eye problem is urgent based on screening questions, and if so, evaluation and management should be in the hospital setting If the patient is not hospitalized at the time of referral, the patient is best referred to the ER or other hospital-based facility equipped to manage both COVID-19 and eye care. CDC or hospital guidelines should be followed for care of COVID-19 patients. Transmission precautions† for treating ophthalmologists include wearing an N-95 mask, gown, gloves and eye protection (face shield or goggles, as above). [Read the American College of Surgeon's guidelines for operating on COVID-19 patients]
[Read the **American College of Surgeons' guidelines** for operating on COVID-19 patients]	
* **Standard** (Universal) **Precautions:** Minimum infection prevention precautions that apply to all patient care, regardless of suspected or confirmed infection status of patient, in any health care setting (e.g., hand hygiene, cough etiquette, use of PPE, cleaning and disinfecting environmental surfaces). See **CDC: Standard Precautions**.	
** Supply permitting, tight-fitting **goggles may be preferable to face shields** for eye protection.	
† Currently, there are national and international shortages of PPE, which also warrant consideration. Excessive use of PPE may deplete the supply of critical equipment required in the future for patients with COVID-19 as the epidemic expands. Use of PPE should be considered on an institutional and case-by-case basis; universal usage for all patient encounters is not appropriate.	
‡ **Transmission Precautions:** Second tier of basic infection control, used in addition to Standard Precautions when patients have diseases that can spread through contact, droplet or airborne routes, requiring specific precautions based on the circumstances of a case. Transmission precautions are required for cases of suspected COVID-19. See **CDC: Transmission-Based Precautions**.	

## 2. Protect health care workers

### Symptoms & when to self-isolate

Symptoms of cough, fever, shortness of breath or flu-like symptoms are typical in COVID-19, but others are also common, including loss of taste and smell.^23^ It is vital to inform patients and health care workers with symptoms of COVID-19 that they should not come to the hospital or clinic, but should self-isolate (i.e., stay at home and avoid contact with other members of the household) for seven days. The United Kingdom's National Health Service (NHS) guidelines state that people who still have a high temperature after 7 days of self-isolation must remain at home until their temperature returns to normal.^24^

**Figure 1:**
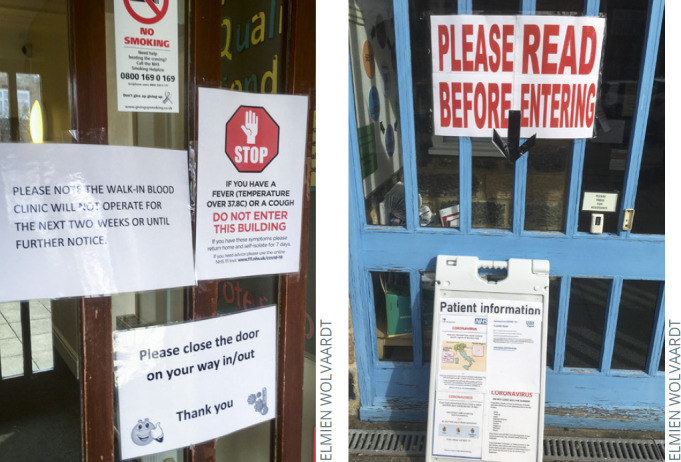
Hospitals and clinics should have clear information for patients to return home and self-isolate if they have symptoms. UK

The incubation period for COVID-19 is thought to range from 1 to 14 days, with symptom onset typically around 5 days; these estimates will be updated as more data becomes available.^4^ **Patients may be infectious before showing any symptoms, and some may show no symptoms at all.** A person who has been in contact with someone known to have COVID-19 should self-isolate for 14 days, until such time as WHO guidance is updated.^4^ It is important to follow national guidelines. Communicate this information to patients as early as possible, so that patients who need to self-isolate do not travel to the unit in the first place. If they do arrive at the unit, there should be very clear information to prevent infection spread; e.g., telling patients to return home and self-isolate (Figure 1).

Table 2 provides more detailed guidance on the type of PPE to be worn. It is important to take note of national guidelines. PPE is likely to include disposable gloves, disposable gown, face respirator mask (when appropriate, e.g., FFP3 or N95) and eye protection. Follow strict PPE wearing and removing procedures (see panel). Dispose of PPE appropriately and carry out thorough room cleaning and air exchange after each patient.

**Table 2:** Royal College of Ophthalmologists guidelines on protective equipment^18^

	Disposable gloves	Disposable plastic apron	Disposable fluid resistant gown	Fluid resistant surgical mask	Filtering face piece respirator	Eye/face protection	Slit lamp breath guard
Performing an aerosol-generating procedures (AGPs)	✓ Single use	✗	✓ Single use	✗	✓ Single use	✓ Single use	✗
High risk acute areas, e.g. theatres where AGPs performed, intensive care unit (ITU), high dependency unit (e.g. ophthalmology review of patient in ITU)	✓ Single use	✓ Single use	✓ Sessional use	✗	✓ Sessional use	✓ Sessional use	✗
Theatres where AGPs not done	✓ Single use	✓ Single use	✓ Risk-assess single use, i.e. use instead of apron if splashes are likely	✓ Single or sessional use	✗	✓ Single or sessional use	✗
Working in inpatient area within two metres eg ophthalmology review of ward patients	✓ Single use	✓ Single use	✗	✓ Sessional use	✗	✓ Sessional use	✓ If using fixed slit lamp
Emergency and acute hospital eye clinics	✓ Single use	✓ Single use	✗	✓ Sessional use	✗	✓ Sessional use	✓
Non-emergency /acute eye outpatients	✓ Single use	✓ Single use	✗	✓ Sessional use	✗	✓ Sessional use	✓
**Single use** = disposal or decontamination of device between each patient/procedure, dispose at end of session							
**Sessional use** = dispose at end of session, e.g. at the end of morning clinic or when leaving the care setting							

Aerosol-generating procedures (AGPs). Aspects relevant to ophthalmology are in **bold**:

- **Intubation, extubation** and related procedures, such as manual ventilation and open suctioning of the respiratory tract
- Tracheotomy/tracheostomy procedures (insertion/open suctioning/removal)
- Bronchoscopy and **upper ENT airway procedures** that involve suctioning
  Upper gastro-intestinal endoscopy where there is open suctioning of the upper respiratory tract
- **Surgery procedures involving high-speed devices**
  Some dental procedures (e.g. high-speed drilling)
  **Non-invasive ventilation** (e.g., CPAP and laryngeal masks)
- High-frequency oscillatory ventilation (HFOV)
- Induction of sputum
- High-flow nasal oxygen

Defer surgical procedures where possible. If surgery cannot be avoided, this should be done under local anaesthesia if possible, taking full precautions. General anaesthesia is an ‘aerosol-generating procedure’ (AGP) which creates a high-risk environment for virus transmission. However, even surgery under local anaesthesia will involve significant exposure between medical personnel and patient and should be considered high risk. If surgery must be undertaken, then full PPE should be used for all patients.

### Self-care and personal protection

- **Wash hands regularly and thoroughly; normal soap is adequate.** Use alcohol-based (>60% ethanol or >70% isopropyl alcohol) hand sanitiser or hand gel if hands are visibly clean and water is not available.
- Staff members and patients/visitors must wash their hands using soap and water or alcohol-based hand sanitiser or hand gel on entering the unit.
- Avoid touching your eyes, nose and mouth.
- If available, use appropriate PPE. Guidelines are constantly being updated; however, there is an increasing recognition of the role of eye protection (Table 2). This is something that may be dictated by health management at a higher (regional/national) level.
- Clinical staff not in uniform who are in close contact with patients should wear surgical scrubs. These should be laundered daily in accordance with the unit's policy on the safe laundering of clinical garments.
- The same surgical mask may be worn for multiple patients to be seen at the slit lamp. However, scrupulous care must be taken not to transmit the virus on the front of the mask via hands or clothes. If using the same mask, do not take on and off between patients and do not allow it to dangle on the chest. Never touch the front of the mask.
- Put on and remove PPE in an order that minimises the potential for self-contamination (see panel).
- The use of a cloth face covering does not replace a surgical mask or respirator, however, they may be better than no covering at all.^29^,^30^

**How to put on and remove personal protective equipment (PPE)**

**Correct order for putting on PPE**

1. Gown/apron
2. Mask/respirator
3. Goggles/face shield
4. Gloves

**Correct order for removing PPE**

1. Gloves
2. Goggles/face shield
3. Gown/apron
4. Mask/respirator
5. Wash hands^25^

**Figure 2:**
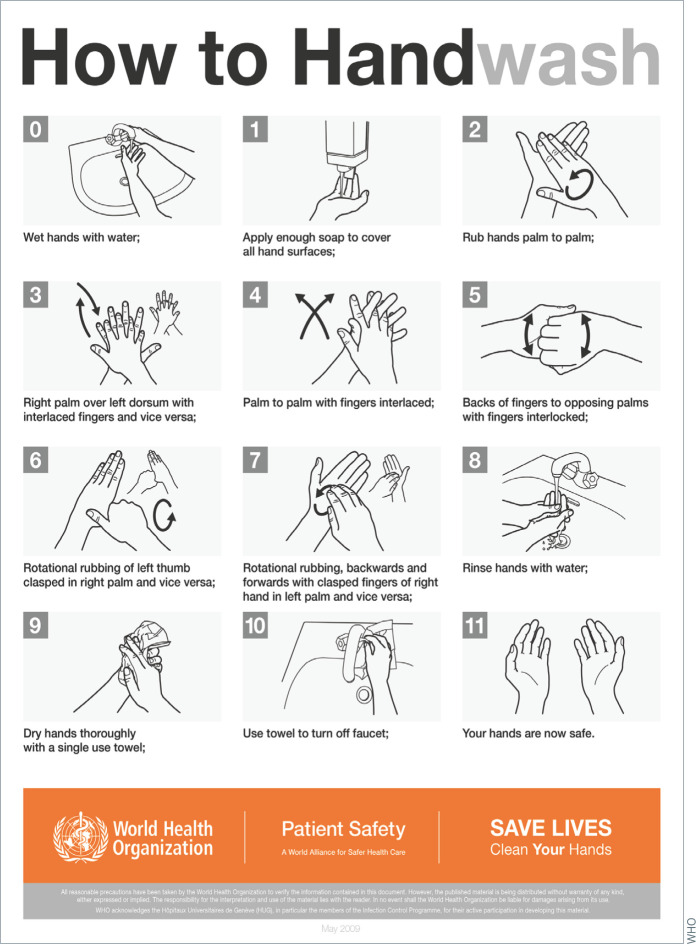
World Health Organization hand washing poster

## 3. Reduce transmission to other patients, and between patients and health care workers

### A. Patient triage

- Patients should already have been notified not to travel to the health care unit if they have symptoms of COVID-19.
- Prominent information posters at the entrance to the unit should alert anyone who arrives to return directly home if they have symptoms and to stay at least 2 metres away from others. If urgent/emergency care is required, they should be directed to an isolation area where they can be examined by health care personnel wearing protective equipment.
- A health care worker should ask patients about possible symptoms on arrival at the booking-in or reception area and direct them appropriately.

### B. Instructions for patients

- Patients and visitors must wash their hands using soap and water alcohol-based (>60% ethanol or >70% isopropyl alcohol) hand sanitiser or hand gel on entering the unit.
- Some countries advise that patients wear masks to limit transmission to medical personnel or other patients.^25–28^
- The use of a cloth face covering does not replace a surgical mask or respirator, however, they may be better than no covering at all.^29^,^30^

### C. While examining the patient

**Figure 3:**
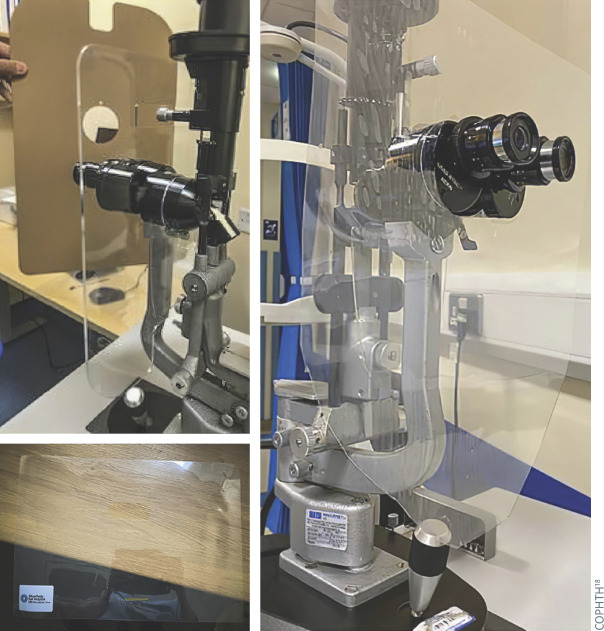
Slit lamp breath guards

- Minimise patient contact time by reviewing the notes beforehand. One suggestion is for the patient to be placed in one room and the clinician in another and for them to talk via a phone or a tablet computer. The clinician then enters the patient room, conducts the examination as swiftly as possible (without talking) and exits the room.
- Avoid shaking hands, or any other patient contact, as much as possible.
- Use slit lamp barriers (breath guards or breath shields), Figure 3. These may be available commercially or they can be made from materials such acetate sheets (used for overhead projectors), clear plastic or Perspex. Cut holes for the slit lamp eyepieces; it may help to use a cardboard template.
- Minimise investigations. For example, perform visual fields and OCT scans only if absolutely necessary. Avoid tonometry if possible.
- Clean surfaces and instruments between patients. Use disposable gloves and a solution of household bleach (1.5 tablespoons per litre of water) or alcohol solutions with at least 70% alcohol.
- Remember to clean patient seats, clinic door handles and phones, if used.
- Remember to clean the breath guard, on/off switch and any controls or buttons when cleaning the slit lamp.

### D. The work environment

- Keep waiting rooms as empty as possible, with preferably at least 2 metres between individuals.
- Prevent overcrowding in the examination room by restricting entry by accompanying persons where possible.
- Routine daily cleaning of the clinic environment with appropriate disinfectants is vital.

**Conjunctivitis**

COVID-19 can cause conjunctivitis and virus particles may be found in ocular secretions.^31^

There is debate as to whether conjunctivitis is a high-risk feature for COVID-19. Although a follicular conjunctivitis can be caused by the virus, this seems to be a relatively uncommon and a non-specific sign that presents later in the clinical course. However, a health care worker who was part of an expert task force to visit Wuhan developed conjunctivitis as the first symptom of COVID-19 despite being fully gowned with protective suit and N95 respirator.^32^ Health care workers have subsequently been urged to wear eye protection when in close contact with patients; this is also reflected in recently updated guidance.^15^,^33^ Many cases of conjunctivitis do not actually require review and it is important that patients with possible symptoms of COVID-19 are instructed to self-isolate unless they require hospital in-patient care. If a patient does present with any conjunctivitis, it would seem sensible to wear full PPE until more is known.

**Reusing eye protection**

The CDC acknowledges that eye protection is often reusable, not disposable, and provides the following guidelines on re-use.^34^

When manufacturer instructions for cleaning and disinfection are unavailable, consider these steps:

1. While wearing gloves, carefully wipe the *inside, followed by the outside* of the face shield or goggles using a cleaning wipe or a clean cloth saturated with pH-neutral detergent solution.
2. Use a wipe or clean cloth saturated with a registered hospital disinfectant solution to wipe the outside of the face shield or goggles carefully.
3. Wipe the outside of face shield or goggles with clean water or 70% alcohol to remove residue.
4. Fully dry (air dry or use clean absorbent towels).
5. Remove gloves and perform hand hygiene.
